# Do bony pelvis parameters affect perioperative outcomes in open radical prostatectomy?

**DOI:** 10.1016/j.prnil.2022.05.002

**Published:** 2022-05-26

**Authors:** Serdar Kalemci, Kasim E. Ergun, Fuat Kizilay, Alp Akyol, Adnan Simsir

**Affiliations:** Department of Urology, Ege University Faculty of Medicine, Izmir, Turkey

**Keywords:** Bony pelvic, Prostate cancer, Radical prostatectomy, Vesicourethral anastomosis

## Abstract

**Objective:**

The present study aimed to evaluate the predictive value of bony pelvic parameters measured by computerized tomography (CT) for use in the estimation of the likely technical difficulties that may be encountered when performing open radical prostatectomy (RP) for localized prostate cancer.

**Material and methods:**

One hundred patients, undergoing open RP for localized prostate cancer, were evaluated between October 2016 to November 2018. All operations were performed by the same experienced surgeon. Pelvic parameters were measured using spiral CT images. Data were retrospectively collected from medical, operative, radiology, and pathology records and analyzed. Positive surgical margin (PSM), presence of vesicourethral anastomosis stricture (VUAS) and urine leakage, operative time, urethral catheterization time, and estimated blood loss were used as indicators of operative difficulty. Univariate and multivariate analyses were performed to determine the significance of these variables.

**Results:**

There was no significant correlation between the pelvic parameters of the patients and the presence of PSM, VUAS, and urine leakage. Only PSA levels and pathological tumor stage were higher in patients with PSM (p = 0.002 and p = 0.001). On univariate and multivariate analyses, none of the individual pelvic parameters assessed showed a significant relationship with the operation time, estimated blood loss, and urethral catheterization time. In univariate analysis, there was a significant relationship between PSA levels and pathological tumor stage and operation time (p = 0.048 and p = 0.001, respectively).

**Conclusion:**

Bony pelvic parameters may not be a significant factor in influencing the perioperative outcomes of open RP. Higher PSA levels and pathological tumor stage may lead to surgical margin positivity and longer operative time.

## 1 Introduction

Prostate cancer is the second most commonly diagnosed cancer in men and the fifth most common cause of death globally.^1^ The standard treatment for most patients having clinically localized prostate cancer is radical prostatectomy (RP). The goal of RP, regardless of approach, is to eradicate cancer while preserving pelvic organ functions whenever possible.^2^ RP can be performed by open, laparoscopic, or robot-assisted techniques. Although open surgery is still common for managing prostate cancer, surgical approaches have evolved from open to laparoscopic and robotic-assisted procedures. There is no evidence to support the comparative effectiveness of laparoscopic RP or robot-assisted RP for oncological outcomes when compared to open RP.^3^ Although analyses of large databases show that the popularity of robot-assisted RP is increasing rapidly,^4^ open RP surgery is still performed worldwide due to the cost of the robot-assisted procedure. Compared to surgeries performed in the abdominal cavity, pelvic surgeries are more difficult due to bone restriction, a deep and narrow working area, and a poor field of view. Thus, open RP is a challenging procedure due to the deep location of the prostate in the pelvic cavity. A narrow or small pelvis, as well as a deeply located prostate, may necessitate a difficult operation and increase the risk of perioperative and postoperative complications.

Pelvimetry, which is the radiological measurement of pelvic bone dimensions, has been performed for many years. The genesis of the measurement of pelvic size was originally intended for the prediction and assessment of cephalopelvic disproportion in obstetric practice. In urology practice, the patient's bony pelvic parameters are important, as RP is performed in an area where movement is restricted, such as the pelvic cavity, which makes visualization and freedom of motion complicated. It has been shown in a previous study that bony pelvis parameters have an influence on the results of open RP.^5^ Similarly, it has been shown that resection margin positivity in rectal cancer surgery can be predicted by preoperative pelvic measurements.^6^

We hypothesized that performing open RP in patients with a narrow and small pelvis would make the surgery technically more difficult so that these patients may have a positive surgical margin (PSM) and vesicourethral anastomotic stricture (VUAS) may develop due to difficulties in vesicourethral anastomosis step of the procedure. In addition, we hypothesized that these patients may have more blood loss during the operation, prolonged urine leakage from the vesicourethral anastomosis in the postoperative follow-up, and longer operative and urethral catheterization times. At present, there is no consensus on how bony pelvic parameters influence the technical difficulty of performing open RP for localized prostate cancer, and there is a lack of reliable evidence analyzing the association between pelvic anatomy and operation outcomes. The main aim of this retrospective study was to assess the impact of preoperative measurement of bony pelvic parameters on the perioperative outcomes of open RP for localized prostate cancer.

## 2 Materials and Methods

### 2.1 Patient selection and data collection

One hundred forty-seven patients who underwent open RP for localized prostate cancer between October 2016 and November 2018 were included in the study. A total of 47 patients were excluded from the study for the following reasons: 28 patients did not have an adequate radiological evaluation, 10 patients did not have sufficient data, and 9 patients had a history of pelvic surgery or trauma and radiotherapy. Therefore, 100 patients who had adequate computerized tomography (CT) imaging to allow measurement of bony pelvic measurements and who underwent open RP were included in further analysis. All procedures were performed by a single surgeon with high experience and volume (approximately 800 open RP and 300 robot-assisted laparoscopic RP). Patient data were retrospectively collected from patient files and records. Age of the patients, prostate volume, preoperative PSA levels, body mass index (BMI), operative time, estimated blood loss, length of stay and urethral catheterization, postoperative data (pathological tumor stage, Gleason score, PSM, and lymph node positivity), follow-up time, and presence of VUAS and urine leakage were recorded and evaluated.

### 2.2 Evaluation criteria

RP specimens of 100 patients were evaluated by two experienced genitourinary pathologists. The following parameters were analyzed in pathological evaluation: prostate volume, pathological tumor stage, Gleason score, extraprostatic extension, seminal vesicle invasion, surgical margin status, and lymph node metastasis. The 2016 Tumour, Node, Metastasis (TNM) classification of the International Union Against Cancer is used for clinical staging and classification of prognostic groups.^7^ Patients suffering from straining to urinate, urinary frequency, poor stream, and incomplete bladder emptying in the postoperative period were evaluated for VUAS. A definitive diagnosis was made by performing urethroscopy in patients with low Qmax and obstructive flow patterns in the uroflowmetry test. Patients with persistent laboratory-confirmed urine from the pelvic drain in the postoperative follow-up were evaluated as prolonged urinary leakage. The main outcome measures of technical difficulty during open RP were the presence of a PSM, prolonged urine leakage, and a detected VUAS in postoperative follow-up.

### 2.3 Pelvimetry

**Figure 1:**
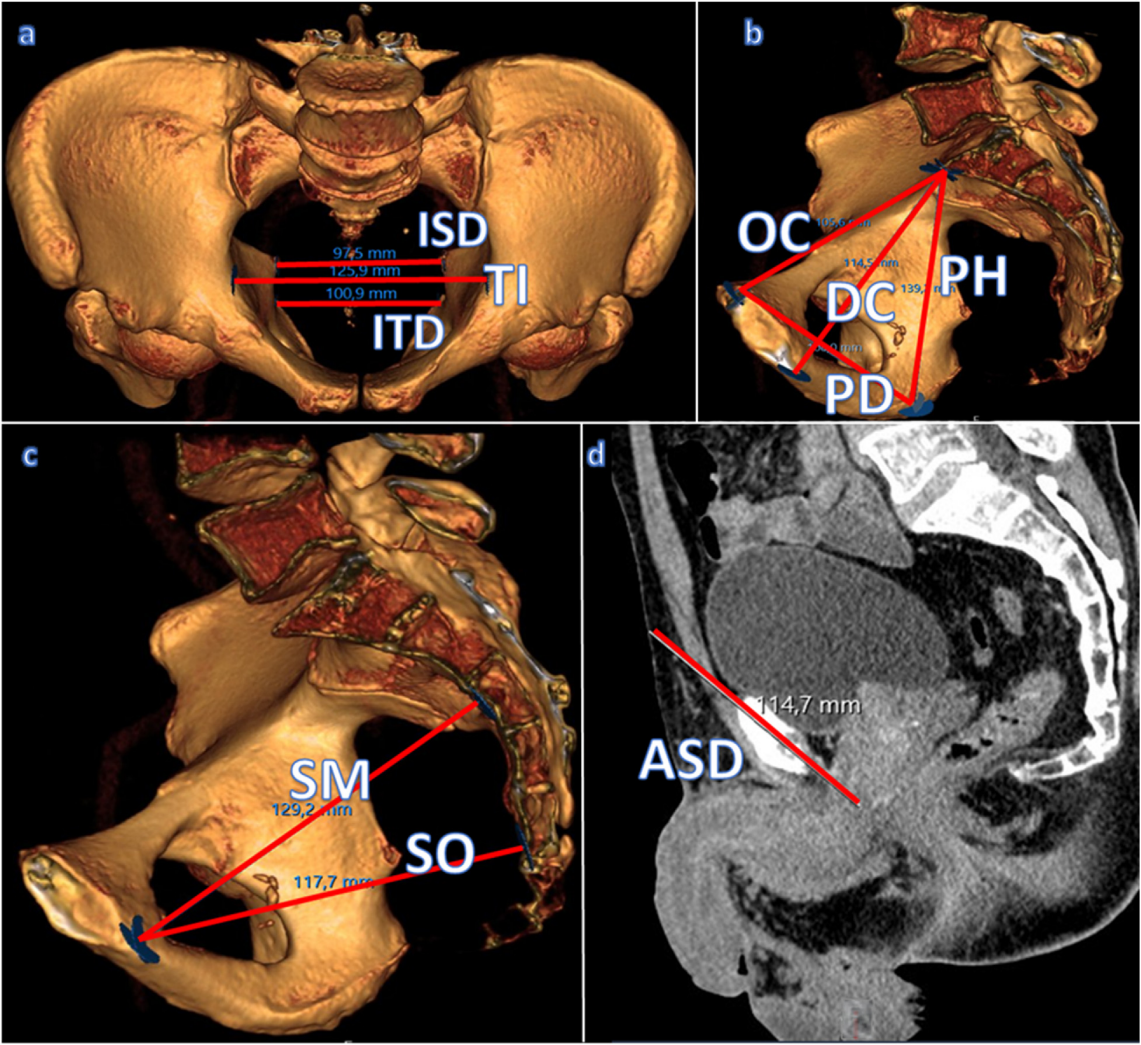
Pelvic parameters measured with CT scan; (a) interspinous distance, transverse inlet, and intertuberous distance; (b) obstetric conjugate, pelvic height, pelvic depth, and diagonal conjugate; (c) sagittal midpelvic and sagittal outlet; (d) apex-skin distance.

All patients underwent thoraco-abdominopelvic CT in the same center. Examinations were performed using a General Electric 64-Slice Discovery HD750 CT Scanner (Boston, Massachusetts, ABD). For standardization, only CT scans performed with the device in our center were included in the study. All measurements were made by a single observer who was blinded to all clinical information. Pelvic dimensions were obtained using midsagittal and axial sections of the pelvis. The bony pelvic parameters recorded are defined in Table 1 and represented graphically in Fig. 1. The pelvic cavity index (PCI) was calculated by the formula as previously described.^8^ PCI was described as OC (distance from the most superior aspect of the pubic symphysis to sacral promontory) X ISD (shortest distance between spinous processes)/PD (shortest distance between spinous processes).

**Table 1:** Definitions and mean bony pelvic parameters measured by CT pelvimetry.

Measurement	Definition	Mean bony pelvic parameters
Transverse inlet (TI)	Widest transverse pelvis brim distance	12.3 (10.2–15.1)
Interspinous distance (ISD)	Shortest distance between spinous processes	9.7 (8.2–12.3)
Intertuberous distance (ITD)	Widest distance between the inferomedial aspects of the ischial tuberosities	10.5 (7.7–13.7)
Obstetric conjugate (OC)	Shortest distance from promontorium to the superior aspect of the symphysis	11.2 (8.9–13.4)
Pelvic height (PH)	Distance between promontorium and ipsilateral tuber ischiadicum	14.7 (11.1–17.2)
Pelvic depth (PD)	Distance between the superior aspect symphysis and ipsilateral tuber ischiadicum	11.6 (8.5–14.8)
Sagittal outlet (SO)	Inferior inner aspect of the symphysis to sacrococcygeal junction	10.6 (8.9–12.8)
Sagittal midpelvic (SM)	Inferior inner aspect of the symphysis to the sacrum along the plane of the spinous process	12.2 (10.1–14.6)
Diagonal conjugate (DC)	Inferior aspect of the symphysis to promontorium	14.2 (11.1–16.9)
Apex-skin distance (ASD)	Distance between the apex of the prostate and the midpoint of the median inferior incision	14.7 (10.5–20)

### 2.4 Open retropubic radical prostatectomy technique

Under general anesthesia, after the patient was placed in a supine position with overextension of the pelvis, 18 FR Foley catheter was introduced, and a lower midline incision was performed through the skin. Subcutaneous tissue and muscles were cut through the suprapubic incision, and Retzius space is exposed. After the adjacent fat tissue is removed, the endopelvic fascia was incised medially, and the dorsal venous plexus was ligated with 0 Vicryl suture and cut. The urethra was released, suspended, and cut. The prostate was released by sharp and blunt dissections from the Denonvilliers’ fascia. Bilateral ductus deferens were clamped and cut, and the right and left seminal vesicles were removed en bloc with the prostate. A 22 FR Foley catheter was introduced in the bladder. The vesicourethral anastomosis was performed from six foci with 2/0 monocryl sutures. One Jackson-Pratt® drain was inserted into the surgical area, and the fascia and skin were closed with 0 PDS suture and stapler, respectively.

### 2.5 Statistical analysis

Statistical analyses were performed using the SPSS software program, version 20.0 for Windows (SPSS, Inc., Chicago, IL, USA). PSM, VUAS, prolonged urine leakage, estimated blood loss, operative time, and urethral catheterization time were defined as dependent variables, and pelvic anatomical and clinicopathological parameters were defined as independent variables. Determination of statistically significant factors was made by univariate and multivariate analysis. Statistical analyses were carried out using an unpaired *t*-test and the Mann–Whitney *U*-test for univariate analysis. Multivariate analyses were performed using a multiple linear regression model with a backward method and multivariate logistic regression. A p-value of less than 0.05 was considered to be statistically significant.

## 3 Results

In total, 100 patients diagnosed with clinically localized prostate cancer and who underwent open RP were included in the study. The mean patient age was 65.9 (43–80) years, and BMI was 26.4 kg/m^2^ (19.1–32.6). The mean preoperative serum PSA level was 12.7 ng/dL (1.6–66.4), and the prostate volume was 54 g (15–120). The patients’ characteristics are listed in Table 2. The mean (range) bony pelvic dimensions, as evaluated on CT: Transverse inlet 12.3 (10.2–15.1) cm, interspinous distance 9.7 (8.2–12.3) cm, intertuberous distance 10.5 (7.7–13.7) cm, obstetric conjugate 11.2 (8.9–13.4) cm, pelvic depth 11.6 (8.5–14.8) cm, pelvic height 14.7 (11.1–17.2) cm, sagittal outlet 10.6 (8.9–12.8) cm, sagittal midpelvic 12.2 (10.1–14.6) cm, diagonal conjugate 14.2 (11.1–16.9) cm, apex-skin distance 14.7 (10.5–20) cm, and PCI index 7.7 (6.2–11.2). There was no significant relationship between any of the assessed pelvic dimensions and other variables, including age, PSA level, BMI, or prostate volume.

**Table 2:** Patients’ characteristics

Variable	Value
Mean age, year	65.9 (43–80)
Mean prostate volume, g	54 (15–120)
Mean PSA level, ng/dL	12.7 (1.6–66.4)
Gleason score, n (%) 6 7 ≥8	25 (25) 57 (57) 18 (18)
Pathological stage, n (%) T2 T3a T3b	45 (45) 28 (28) 27 (27)
Mean catheterization, day	18.1 (15–35)
Mean length of stay, day	6.6 (3–32)
Mean operative duration, min	146 (110–220)
Positive surgical margin, n (%)	34 (34)
VUAS, n (%)	12 (12)
Mean estimated blood loss, mL	385 (175–1240)
Mean follow-up, month	39.2 (21–53)

A negative surgical margin was achieved in 66% (66 of 100) of the patients. Only five of 34 patients with PSMs had the organ-confined disease (pT2c). The pathological tumor stage was determined as T3 in the remaining 29 patients. For surgical margin positivity, no pelvic parameter had a significant effect on univariate analysis. PSA and pathological tumor stage were the only variable that was significantly associated with PSM on both univariate and multivariate analyses (p = 0.002 and p = 0.001, p = 0.012 and p = 0.007, respectively). The univariate analysis of predictors of PSM and VUAS is shown in Table 3. VUAS was detected in 12% of patients (12 of 100) at a mean follow-up of 39.4 months. None of the variables and pelvic parameters measured demonstrated significant associations with VUAS on univariate and multivariate analysis. In the postoperative follow-up, urinary leakage was observed in 6% of the patients. There was no significant association between parameters and urine leakage in univariate and multivariate analysis. Multivariable logistic regression analysis of factors predictive of PSM, VUAS, and urine leakage is shown in Table 4.

**Table 3:** Analysis of pelvic parameters, age, PSA levels, BMI in relation to PSM and VUAS (Note: PV: prostate volume, PCI: pelvic cavity index, BMI: Body mass index)

Variable (mean, range)	PSM (−) n = 66	PSM (+) n = 34	p-value	VUAS (−) n = 88	VUAS (+) n = 12	p-value
**Age**	65.3 (43-77)	67 (53-80)	0.178	65.9 (43-80)	65.7 (53-77)	0.890
**PSA**	9.5 (1.6-33.9)	18.8 (3.9-68)	0.002	12.2 (1.6-39)	14.6 (3-68)	0.870
**BMI**	26.6 (20.9-32.6)	26.3 (19.1-28.8)	0.747	25.9 (20.9-30.7)	26.8 (21.7-32.6)	0.704
**PV**	54.5 (15-120)	54.3 (20-100)	0.856	53.4 (20-120)	58.2 (15-110)	0.509
**TI**	12.3 (10.5-13.4)	12.3 (10.2-15.1)	0.915	12.3 (10.2-15.1)	12.4 (11-14.1)	0.614
**ISD**	97.9 (8.5-12.3)	97.6 (8.2-11.9)	0.879	9.7 (8.2-12.3)	9.7 (8.5-11.9)	0.825
**ITD**	10.5 (8.2-13.7)	10.6 (7.7-12.4)	0.209	10.6 (8.4-13.7)	10 (7.7-11.8)	0.112
**OC**	11.3 (8.9-13.4)	11.2 (9.6-13.3)	0.769	11.3 (8.9-13.4)	11.1 (9.9-13.3)	0.164
**PD**	11.6 (9.7-14.8)	11.5 (8.5-12.9)	0.813	11.7 (9.9-14.8)	11.2 (8.5-12.5)	0.056
**PH**	14.6 (11.1-16.9)	14.9 (13.2-17.2)	0.200	14.7 (11.1-17.2)	14.6 (12.3-16.7)	0.599
**SO**	10.6 (9.2-12.6)	10.5 (8.9-12.8)	0.687	10.6 (8.9-12.8)	10.4 (8.9-12.2)	0.432
**SM**	12.3 (10.4-14.6)	12.1 (10.1-14.4)	0.647	12.2 (10.1-14.6)	12.3 (10.7-14.4)	0.874
**DC**	14.2 (11.7-16.8)	14.2 (11.1-16.9)	0.925	14.2 (11.7-16.9)	14.2 (11.1-16.6)	0.951
**ASD**	14.8 (10.7-20)	14.5 (10.5-19)	0.125	14.7 (10.5-20)	14.8 (11.4-20)	0.911
**PCI**	7.7 (6.2-11.2)	7.7 (6.2-10.1)	0.965	7.7 (6.2-11.2)	7.5 (6.4-9.3)	0.370

**Table 4:** Multivariate logistic regression analysis of factors affecting PSM, VUAS, and urine leakage

Variable	PSM OR (95% CI)	p-value	VUAS OR (95% CI)	p-value	Urine Leakage OR (95% CI)	p-value
Age	0.97 (0.89-1.05)	0.488	1.00 (0.91-1.11)	0.976	1.04 (0.77-1.39)	0.789
PSA	1.08 (1.01-1.12)	0.012	0.96 (0.89-1.02)	0.255	0.92 (0.67-1.25)	0.598
BMI	1.07 (0.94-1.22)	0.277	0.80 (0.66-0.96)	0.819	0.76 (0.47-1.24)	0.282
Tumor stage	1.12 (1.05-1.18)	0.007	0.95 (0.88-1.12)	0.242	1.02 (0.86-1.19)	0.464
Prostat volume	0.99 (0.97-1.02)	0.943	1.00 (0.97-1.03)	0.760	0.99 (0.88-1.11)	0.927
TI	0.95 (0.88-1.03)	0.244	0.96 (0.87-1.06)	0.513	0.85 (0.61-1.19)	0.369
ISD	0.91 (0.42-1.94)	0.813	1.62 (0.55-4.67)	0.373	1.13 (0.16-8.5)	0.798
ITD	1.06 (0.98-1.13)	0.105	0.99 (0.92-1.08)	0.990	1.29 (0.94-1.7)	0.109
OC	0.91 (0.48-1.76)	0.800	1.53 (0.61-3.83)	0.365	0.78 (0.11-3.5)	0.812
PD	1.00 (0.92-1.09)	0.848	0.94 (0.85-1.05)	0.124	1.02 (0.72-1.42)	0.910
PH	0.96 (0.91-1.01)	0.196	1.03 (0.95-1.12)	0.418	1.19 (0.85-1.65)	0.294
SO	1.01 (0.93-1.07)	0.932	1.12 (1.02-1.23)	0.106	1.27 (0.87-1.85)	0.207
SM	0.91 (0.77-1.07)	0.272	0.96 (0.80-1.16)	0.735	1.57 (0.99-2.52)	0.082
DC	1.15 (0.62-2.13)	0.657	0.73 (0.33-1.64)	0.458	0.60 (0.22-3.02)	0.544
ASD	0.98 (0.96-1.01)	0.426	1.00 (0.97-1.03)	0.825	0.96 (0.87-1.06)	0.483
PCI	1.17 (0.45-3.00)	0.742	0.52 (0.13-2.05)	0.354	0.45 (0.29-6.93)	0.568

On univariate and multivariate analysis, none of the individual pelvic parameters assessed showed a significant relationship with the operation time, estimated blood loss, and urethral catheterization time (Table 5). In univariate analysis, there was a significant relationship between PSA and pathological tumor stage with operation time (p = 0.048 and p = 0.001, respectively). Overall, in a univariate comparison and multivariate analysis of pelvic measurements as determined by preoperative imaging, none of the measured pelvic parameters had any significant effect on perioperative outcomes.

**Table 5:** Multivariable linear regression analysis of factors associated with operative time, estimated blood loss, and catheter duration.

Variable	Operative time			Estimated blood loss			Catheter duration		
	β	T	p	β	T	p	β	T	p
Age	0.009	0.094	0.926	0.031	0.282	0.779	−0.172	−1.663	0.100
PSA	0.161	1.607	0.077	0.002	0.023	0.982	−0.064	−0.617	0.539
BMI	0.233	2.293	0.094	−0.091	−0.812	0.419	0.062	0.589	0.557
Tumor stage	0.132	1.324	0.082	−0.088	−0.654	0.091	0.064	1.224	0.214
Prostat volume	0.195	1.828	0.071	−0.216	−1.846	0.068	−0.026	−0.230	0.819
TI	−0.231	−1.861	0.076	−0.043	−0.316	0.753	−0.034	−0.267	0.790
ISD	1.337	1.318	0.191	−0.111	−0.100	0.921	−0.628	−0.595	0.553
ITD	−0.025	−0.182	0.856	−0.081	−0.535	0.594	0.280	1.959	0.093
OC	1.204	1.112	0.269	−0.180	−0.152	0.880	−0.600	−0.533	0.596
PD	0.007	0.053	0.958	0.100	0.693	0.490	−0.200	−1.457	0.149
PH	−0.176	−1.291	0.200	0.073	0.488	0.627	−0.064	−0.449	0.655
SO	0.109	0.951	0.344	−0.094	−0.742	0.460	0.172	1.443	0.153
SM	0.172	0.734	0.465	−0.058	−0.226	0.822	0.360	1.480	0.143
DC	−1.112	−1.002	0.310	0.203	0.170	0.866	0.343	0.303	0.762
ASD	−0.220	−2.113	0.086	0.104	0.920	0.360	−0.105	−0.977	0.331
PCI	−1.797	−1.184	0.240	0.144	0.087	0.931	0.659	0.418	0.677

## 4 Discussion

In the present study, we evaluated and reported the influence of bony pelvis parameters on perioperative outcomes in patients undergoing open RP surgery, and we carried out our analyses on a cohort of 100 patients. In addition to pelvic parameters, we analyzed the relativity of parameters, such as age, PSA, prostate volume, BMI, and pathological tumor stage with perioperative outcomes. Evaluation of VUAS, as well as PSM in our study, makes it different from previous studies. The major surgical treatment for clinically localized prostate cancer is RP, which provides long-term oncological control. Although robot-assisted RP is becoming more widely used,^9^ a large proportion of patients are still managed with open RP in many countries due to financial constraints.^10^ Regardless of the method of the procedure, RP is considered one of the most difficult pelvic procedures due to the anatomical position of the prostate in the bony pelvis and being oncological as well as being a reconstructive surgery.

The prediction of the difficulty of pelvic dissection is important in planning on the choice of operation. For many years, bony pelvis characteristics and angle measurements have been employed particularly in the obstetrics practice. The goal of pelvimetry in women whose fetuses have a cephalic presentation is to detect the presence of cephalo-pelvic disproportion and thus the requirement for cesarean section. With the advances in radiology, pelvimetric measurements which were initially made with X-ray began to be made with CT and magnetic resonance imaging (MRI) later on.^11^ Patients with prostate cancer usually require spiral CT enhanced scanning for preoperative staging evaluation. When compared to MRI, spiral CT offers high sensitivity and specificity while also being relatively affordable. Thus, CT pelvimetry may be used expediently in patients with prostate cancer. CT pelvimetry is also an accurate and reliable technique for obtaining pelvimetric measurements, which has been utilized in patients with rectal cancer. For many years, CT pelvimetry has been also used in patients with rectal cancer as an accurate and reliable method.^12^ Pelvic parameters vary from one person to another depending on weight, height, race, and bony pelvis structure. However, it was observed that the bone pelvis parameters that we measured in our study were similar to the common parameters measured in the patient population in a study conducted in Korea.^8^ In patients with narrow bony pelvis, surgery can be challenging due to limited surgical mobility, insufficient working area and deficient vision, leading longer operating times. In our study, surgical difficulty was demonstrated by the presence of PSM, VUAS, urine leakage, prolonged operative, and urethral catheterization time, and estimated blood loss.

Our study focused on precisely defining the relationship between pelvic parameters and removal of the prostate in line with its anatomy. Based on previous studies similar to our study, in a study investigating the impact of pelvic parameters on surgical outcomes, it was stated that pelvic parameters measured by MRI affected these results, but no statistically significant difference was observed.^5^ While it was seen that PSA levels and pathological tumor stage had a significant effect on PSM in our cohort, in this study, only PSA levels and BMI were found to be effective, although not significant for PSM. In a study where measurements were made with MRI and especially apical PSM was evaluated, the parameter measured as apical depth was shown to be an independent predictor, and it was highlighted that the location of the prostate in the pelvis may influence the complexity of surgery.^13^ In a large cohort that analyzed medical and surgical complications in patients undergoing both open and laparoscopic RP, it was reported that none of the variables significantly predicted complications.^14^ Interestingly, in a study investigating the effect of narrow pelvis structure in patients who underwent robot-assisted RP, it has been shown that a deep and narrow pelvis increases the risk of a PSM as well as the surgical time.^15^ In addition, this study was conducted to emphasize the importance of the surgeon's experience in the surgery of patients with a narrow pelvis and suggested that deeper pelvises, regardless of surgeon experience, may restrict adequate or desired wrist articulation during robot-assisted RP.

The vesicourethral anastomosis in RP surgery is one of the most critical and challenging parts in terms of affecting the patient's quality of life after the surgery.^16^ Failure to achieve a watertight anastomosis is associated with postoperative urine leakage, VUAS, and delayed urinary continence.^17^ We can base on the lack of a significant relationship between the pelvic measurements and VUAS and urine leakage to the fact that all surgeries were performed by a surgeon with experience in this field, and the anastomosis technique made from six foci was used as a standard in the surgery. We believe that the experience of the surgeon is a crucial factor in outcomes in these technically challenging procedures such as performed in patients with a narrow pelvis. In addition to the vesicourethral anastomosis, control and ligation of the dorsal vein complex have a crucial role in open RP surgery. In patients with a narrow or deep pelvis and large prostate, needle passage can be challenging during dorsal vein complex control. Inaccurate ligation of DVC may lead to severe bleeding, improper apical dissection, VUAS, and postoperative incontinence.^18^ Thus, accurate ligation of the dorsal vein complex aids in minimizing blood loss, keeping the surgical field clear, and preventing the development of related complications. In our study, none of the measured pelvic parameters were an independently significant factor affecting the operative time, EBL, or urethral catheterization time. These results are consistent with a prior study that has suggested that none of the pelvic parameters were associated with operative time and estimated blood loss.^5^

Our study has several limitations. First, the retrospective nature of the study. Second, the inclusion of cases performed by a single surgeon for standardization of the study may have affected the results. Different results could have been obtained with a surgeon with less volume and experience. Third, the relatively low number of cases and the fact that we randomly selected the cases from among the patients with medical records in our institute may have caused selection bias.

## 5 Conclusion

In the present study, we evaluated the impact of anatomical dimensions, measured from preoperative CT affecting perioperative outcomes after open RP. We concluded that bony pelvic parameters may not be a significant factor in influencing the perioperative outcomes of open RP. Even though RP can be laparoscopically or robot-assisted done in recent years, open RP is still a valid option for these patients. In patients with locally advanced disease and a difficult pelvis structure, it may be necessary to change the way the surgery is performed to obtain better results. Therefore, studies with larger sample sizes and well-designed control groups need to be conducted for more precise outcomes, and a certain predictor of the complexity of pelvic dissection may aid in the decision of whether to perform the RP open, laparoscopic, or robot-assisted approach.

## Grant support & financial disclosures

None.

## Conflicts of interest

All authors have no conflict of interest to declare.
